# Kelsey’s Improved Vulcanizer

**Published:** 1870-01

**Authors:** 


### KELSEY'S IMPROVED VULCANIZER.

This apparatus invented by Dr. C. M. Kelsey, of Mt. Vernon, Ohio, has for its object a rapid and efficient method of vulcanizing, with *absolute* freedom from danger by explosion. These are accomplished by the use of the sand bath, in the properly constructed and arranged apparatus.

Considering the great number of accidents that have occurred, and many of them of a very serious character too, with the ordinary steam vulcanizer, we regard this as quite an important invention, one that all those who use rubber for dental purposes will prize highly.

The Dr. recently demonstrated the working of his apparatus to the profession in this city, and gave instructions to the classes in the Dental College; and so well pleased were they that they gave him the following evidence of appreciation which we think was justly due the Doctor.

At a meeting of the Students of the Ohio Dental College, on Friday, Jan. 7th, 1870, Mr. Porter was appointed chairman of a committee on resolutions, due Dr. C. M. Kelsey.

*Resolved*, That the students of the Ohio Dental College are under many obligations to Dr. C. M. Kelsey, for his kindness in demonstrating to us his new process of vulcanizing rubber for Dental and other purposes, and for attaching rubber to metal plates having a burnished surface, by the use of a sand bath.

*Resolved*, That after seeing work done by this new process, we are firmly convinced that it is superior to the old process of vulcanizing, as the work presents a better color—is cleaner, less liable to break, and is attended with no danger of explosions which so often occur with steam vulcanizers.

*Resolved*, That as better results can be secured by this than by any other process, we join in recommending it to all members of the profession who desire an improvement in this direction, believing its merits will recommend it.

J. M. Porter,

E. C. Moore,

Alex. Warner,

H. K. Lathrop, Jr.

W. Vanantwerp,

} *Committee.*

